# “No patient should die of PPH just for the lack of training!” Experiences from multi-professional simulation training on postpartum hemorrhage in northern Tanzania: a qualitative study

**DOI:** 10.1186/s12909-017-0957-5

**Published:** 2017-07-14

**Authors:** Signe Egenberg, Bjørg Karlsen, Deodatus Massay, Happiness Kimaro, Lars Edvin Bru

**Affiliations:** 1 RN/M PHD, Department of Obstetrics and Gynecology, Stavanger University Hospital, Armauer Hansensv. 20, 4011 Stavanger, Norway; 2 Professor, Department of Health Studies, University of Stavanger, Stavanger, Norway; 3 Research assistant, Mbulu, Tanzania; 4 RN/M, Children’s Department, Kilimanjaro Christian Medical Centre, Moshi, Tanzania

**Keywords:** Qualitative, Multi-professional, Simulation training, Postpartum hemorrhage, Learning features, Teamwork, Confidence

## Abstract

**Background:**

Postpartum hemorrhage (PPH) is a major cause of maternal morbidity and mortality. In Tanzania, PPH causes 25% of maternal deaths. Skilled attendance is crucial to saving the lives of mothers and their newborns during childbirth.

This study is a follow-up after multi-professional simulation training on PPH in northern Tanzania. The purpose was to enhance understanding and gain knowledge of important learning features and outcomes related to multi-professional simulation training on PPH.

**Methods:**

The study had a descriptive and exploratory design. After the second annual simulation training at two hospitals in northern Tanzania, ten focus group discussions comprising 42 nurse midwives, doctors, and medical attendants, were carried out. A semi-structured interview guide was used during the discussions, which were audio-taped for qualitative content analysis of manifest content.

**Results:**

The most important findings from the focus group discussions were the importance of team training as learning feature, and the perception of improved ability to use a teamwork approach to PPH. Regardless of profession and job tasks, the informants expressed enhanced self-efficacy and reduced perception of stress. The informants perceived that improved competence enabled them to provide efficient PPH management for improved maternal health. They recommended simulation training to be continued and disseminated.

**Conclusion:**

Learning features, such as training in teams, skills training, and realistic repeated scenarios with consecutive debriefing for reflective learning, including a systems approach to human error, were crucial for enhanced teamwork. Informants’ confidence levels increased, their stress levels decreased, and they were confident that they offered better maternal services after training.

## Background

According to the World Health Organization [1], the right to health includes the right to appropriate quality health care that is timely, acceptable, and affordable. Skilled attendance at every childbirth is the most important step in saving the lives of mothers and their newborns [2] and implies support of the normal progression of labour, prevention of complications, and proper and timely interventions if complications do develop. Postpartum hemorrhage (PPH) is one of the major maternal threats that health care providers face regularly; it is a major cause of morbidity and mortality in low-resource countries [3] and causes maternal morbidity in high-resource countries [4].

Tanzania had a fertility rate of 5.1 in 2015. The figures from 2015 indicate that 49% of live births in Tanzania were attended by skilled birth attendants [5]. Tanzania aims to reduce maternal deaths from 410 per 100,000 live births in 2013 to 140 per 100,000 live births by 2030 [6].

PPH is thought to be preventable in most cases [7]. The most important step in preventing maternal morbidity and mortality due to PPH is skilled attendance at birth and access to emergency obstetric care [6]. Skilled attendance includes active management of the third stage of labour, such as administration of oxytocin to the mother soon after childbirth, controlled cord traction, delayed clamping of the cord, and uterine massage [8]. Health authorities and their development partners have implemented a variety of training courses to enhance education and health workers’ competence. Courses such as Emergency Obstetric and Newborn Care (EmONC) and Advanced Life Support in Obstetrics (ALSO) involving mid- and high-level health care providers have been established globally, including Tanzania, during the last 15-20 years [9, 10]**.** These courses are combining evidence-based theory and hands-on training. Regarding EmONC courses, transfer of learning to clinical practice is evaluated and follow-up visits implemented [9]. Scenarios and debriefing sessions are traditionally not part of ALSO and EmONC courses.

### Summary of existing literature

A review on medical education during three decades by Issenberg et al. evaluated the features and use of high-fidelity medical simulation that resulted in effective learning [11]. The evidence suggested that feedback, repetitive practice, curriculum integration, range of difficulty level, multiple learning strategies, capturing of clinical variation, controlled environment, individualized learning, defined outcomes and simulator validity have shown to facilitate learning. Another review by McGaghie et al. 9 years later, based on four decades of simulation-based medical education, summarized 12 features and best practices for optimal effect of training, supporting the main findings of the previous review [12]: feedback, deliberate practice, curriculum integration, outcome measurement, simulation fidelity, skill acquisition and maintenance, mastery learning, transfer to practice, team training, high-stakes testing, instructor training, and educational and professional context. A review on team training on obstetric emergencies showed that simulation training can improve performance and reduce errors and risks by providing an opportunity to train on uncommon and complicated events as teams in a safe environment, and use repetition for increased learning [13]. In situ simulation training was found to enhance health workers’ skills and level of efficacy. Besides, team training in the actual environment contributed to identification of potential hazards and deficiencies within the clinical setting [14]. Moreover, an intervention study in the United States showed improved response times related to PPH management among experienced clinical teams. This was an important finding, because time delay in identification and treatment of PPH can cause a deterioration of the mother’s condition within minutes [15]. Simulation training could help improve response times.

Simulation training, which allows health workers to train on skills and drills without harming the patient, has become the preferred tool for team training to enhance patient safety [16, 17]. However, the success of simulation training is likely to depend on how the design of the training takes into account salient learning features. Realism provides the necessary context for understanding, provided the operator/simulator has face validity. According to Issenberg et al., high degree of realism is important as an approximation of simulation to obstetric emergencies [11]. Bridging theory and practice through simulation training is partly dependent on comportment, in which the teacher or patient actor engages as a real-world role model [18].

A crucial element for learning is feedback [19], which is believed to be the single most important feature of effective learning through scenario-based training [11, 12]. Receiving performance feedback at the team level can enhance the sense of collective efficacy more strongly than if feedback is given individually [20, 21]. By enabling evidence-based practice in a simulated context, changes in performance and culture can be achieved and sustained [22]. Debriefing multi-professional teams with rigorous feedback can strengthen the collective efficacy and teamwork [23]. Health care providers who identify the strengths of collaborative practice in order to manage complex health issues can contribute to improved health services [24]. However, a recent meta-analysis of team training concluded that feedback as such can decrease performance if attention is focusing on personal-related issues more than the training tasks. Feedback is a powerful tool and should be exercised with caution to ensure a safe environment for learning [25]. Feeling psychologically safe is therefore a prerequisite for multi-professional team members to critically reflect and challenge their own habits and beliefs [26].

Research that gives us more insight into the underlying mechanisms of improved outcomes after context-specific, multi-professional training is needed [27]. This study aims partly at elucidating important learning features of effective simulation training by drawing on social cognitive theory. According to social cognitive theory, there is a substantial difference between possessing knowledge, skills, and attitudes and actually using them in demanding situations [28]. To enhance self-efficacy, four factors are crucial: mastery experience; observation of role models mastering tasks, which can serve as vicarious or indirect experience; social persuasion by feedback, in which mastery experiences contrasting or supporting previous experiences are confirmed; and psychological state while under pressure, during which anxiety can be perceived as an energizer [29]. Simulation training may affect self-efficacy, thereby influencing performance through all of these mechanisms.

However, building efficient teams remains a challenge. A study reporting pediatric residents’ experiences with multi-professional simulation training described performance anxiety and debriefing limitations, partly perceived as “positional power”, among young doctors in a hierarchical system, with more experienced nurses having “expert and informational power”. According to the authors, acknowledgement of existing barriers to collaborative practice should be addressed in education and team training [30]. A sociological approach in which power relationships (“sociological fidelity”) are taken into account can enhance transferability from simulation training to multi-professional, clinical practice [31]. Multi-professional training can be used to explore real-life tensions between different cadres and to address boundaries during debriefing [32]. To explore the educational role of collaborative practice or team training was therefore an important part of this study.

### The intervention setting for the quasi-experimental study

This study followed a quasi-experimental study on multi-professional simulation training in two Tanzanian hospitals that was associated with a reduction in transfusion of blood products after training (submitted for publication). The simulation training included nurse midwives, doctors, and medical attendants. The training, which was conducted in Swahili, had a contextual approach based on the locally established PPH protocol [33] and the learning package Helping Mothers Survive: Bleeding after Birth, in Swahili [34]. After technical skills training on the birthing simulator MamaNatalie®, the small teams practiced realistic and severe PPH scenarios based on three learning goals: PPH prevention, PPH management, and communication. Scenarios and subsequent debriefings were repeated to ensure that all trainees could participate actively twice and observe their fellow colleagues twice. Reflective learning was encouraged through debriefings within the team, focusing on perception of responsibility, guilt, and destiny. Critical exploration facilitated by local faculty in a confidential and safe environment challenged trainees’ habits and beliefs [26, 35]. For some of the staff members, the simulation training complemented previous EmONC and ALSO training.

### The current study

The two research questions guiding the qualitative study, were: 1) How did the participants perceive the learning features of multi-professional simulation training on PPH? and 2) How did the participants perceive the learning outcomes of simulation training on PPH management? The study aimed to enhance our understanding and possibly gain new knowledge on what is considered the most important educational aspects of multi-professional simulation training in order to achieve efficient emergency teamwork among maternity staff.

## Methods

A descriptive and exploratory design with focus group discussions (FGDs) was chosen to collect data on the informants’ experiences with multi-professional simulation training on PPH. FGDs can provide a deeper understanding of social phenomena by using open-ended questions exploring experiences, views, motivations, and beliefs [36, 37]. They obtain the points of view of many individuals in a short period of time during a discussion initiated by the interviewer. The participants can share their experiences with each other and respond to other group members’ experiences [38].

### Recruitment and characteristics of informants

The setting under which the informants worked is relevant to understand the results of the current study. The prior training was implemented at two northern Tanzanian health facilities: a zonal consultant hospital with a high admission rate for patients transferred to the hospital due to complications during childbirth, and a regional referral hospital without an operating theatre for 5 years prior to the current study. Due to the huge workload, recruitment depended on the availability to participate. Of the 42 participants who were available for FGDs and agreed to take part in this study, 69% had participated in the simulation training twice (see Table 1). Among the 42 interviewees, 17 had 0-5 years of working experience, 14 had 6-15 years and 11 had more than 15 years of working experience.

**Table 1:** Description of professions and number of training sessions of the informants

	Data collection	Nurse midwives (n)	Medical attendants (n)	Doctors (n)	Total	Participated in training once (n)	Participated in training twice (n)
Regional referral hospital	Six FGDs	14	4	3	21	7	14
Zonal consultant hospital	Four FGDs	12	5	4	21	6	15
Total (n)		26	9	7	42	13	29

In both hospitals, the FGDs included both nurse midwives and medical attendants due to their related work tasks. We also assumed that medical attendants would speak more freely if participating in FGDs with the nurse midwives only. The assistant medical officers and the medical officers at the regional referral hospital were generalists with considerable clinical experience within obstetrics and gynecology. The doctors were invited to participate in separate groups at both facilities due to their specific work tasks and for convenience.

Depending on availability, the groups consisted of 2-6 informants. All interviewees who agreed to participate gave their verbal consent, which was audio-recorded, and they were guaranteed anonymity. Informants were free to withdraw from the study at any time. Participation was coded for transcription and analysis to ensure anonymity.

### Data collection

Two months after the last simulation training in 2014/15, 10 semi-structured FGDs were carried out at the two hospitals in Swahili (except one FGD for doctors in English). A semi-structured interview guide was used to conduct the FGDs and to achieve a thematic and dynamic discussion by exploration and description of the informants’ experiences during simulation training. The interview guide contained seven questions related to the informants’ experiences with simulation training on PPH and how this training influenced their clinical practice (Appendix 1). The questions were based on Bandura’s Social Cognitive Theory on self-efficacy [39] and the two research questions. The dynamic discussion explored concepts of self-efficacy, which is also understood as confidence in Swahili, and the perceived impact of simulation training on their work in the hospital. The discussions were carried out by a Tanzanian research assistant who was not employed at the actual health facilities but known to the informants through the simulation training. His background as a clinical officer, research assistant, and translator, combined with considerable experience as an interviewer, gave him credibility.

The FGDs lasted from 30 to 75 min, or an average of 54 min per FGD. After being recorded, the discussions were transcribed verbatim and translated from Swahili to English by two translators. Many of the informants used Swahili mixed with some English vocabulary. Some of them used the words “knowledge” and “skills” interchangeably, not fully distinguishing between the two terms. Verbatim translation of these words was based on the passage of the text in order to grasp the contextual meaning.

### Data analysis

The only documentation of the FGDs was the audio-recorded discussions, and the analytical process concentrated exclusively on manifest content that was visible and obvious in the transcriptions of the FGDs. The 10 discussions were imported into Nvivo 11 Pro software [40]. The qualitative content analysis was performed in the following steps [41]: 1) every discussion was considered a unit of analysis, 2) data were reduced to meaning units emerging from the text and in conjunction with themes like confidence, teamwork, and motivational factors reflected in the interview guide using a coding framework of Nvivo nodes, 3) each meaning unit was condensed in order to preserve the core of the content while shortening it, 4) categories were created based on commonalities among the codes, as the most essential part of qualitative content analysis, and 5) the nodes were accumulated into three global categories.

The authors were involved in different steps of the data analysis. Initially, the first author (SE) read the transcribed text several times to gain an understanding of its overall content, while the interviewer (DM) reread the transcripts. The first author coded the transcribed text and meaning units were identified. After accumulation of meaning units, the remaining part of every unit of analysis was checked by the first author for meaning units that could have been overlooked. To ensure optimal categorization and to strengthen the credibility of the analyses, the interviewer checked the codes for correctness to avoid personal bias. Four of the authors (SE, DM, BK, LEB) evaluated the nodes and categories after all relevant meaning units were extracted from the units of analysis. All authors (SE, DM, BK, LEB, HK) discussed the findings and reached consensus. The categories emerging from the content analysis were systematized according to the research questions to explore the perceived learning features and learning outcomes from simulation training.

### Trustworthiness

In order to achieve trustworthiness in this research, we ensured reflexivity, relevance, and validity of the qualitative study by forming an experienced, multi-professional team of co-authors [42]. Among some of the co-authors, their preconceptions were based on previous and current experience with simulation training, while two of the co-authors had neither previous experience from simulation training nor from research in a low-resource setting like Tanzania. All co-authors considered the constructed knowledge on learning features and learning outcomes as partial and situated to achieve methodological reflexivity as researchers.

Clinical relevance was obtained by using an interview guide exploring the informants’ experiences and how the training influenced their clinical practice. Relevance was also secured by analysis of the findings by a multi-professional group. Relevant theories on learning features formed the basis of the study.

Validity was ensured by interviewing participants who were the focus of the study and by using appropriate methodology, including the interview guide. The FGDs were all conducted within 2 months of the second simulation training, giving the informants the same period of time to process the experiences from the training and their clinical work. At the time of the FGDs, the significant training-related improvements in patient outcomes at the zonal consultant hospital (submitted for publication) were not yet known. The informants’ perceptions of the training were related purely to their experiences with the training and subsequent clinical practice, which made us consider their perceptions of learning features and outcomes valid. The analyses were conducted consecutively after the FGDs to ensure an appropriate selection of meaning units.

By describing the methodological approach for the sake of validity, the transferability of our findings to similar studies in similar settings can be assumed [42].

## Results

The focus group discussions provided complementary data. The answers were in accordance with the interview guide. Realizing that the interviewees’ previous experiences were partly based on difficult and traumatic events, we found rich descriptions emphasizing their stress level during previous PPH-events.

Three main categories emerged from the data analysis: the first main category *Educational aspects of the simulation training* was in accordance with the first research question on learning features, and the other two categories *Perceived competence* and *Motivational and emotional aspects* were in accordance with the second research question on learning outcomes. All main categories included sub-categories and are summarized in Table 2. References to direct quotations in the text refer to informants: registered nurse midwives (RN/M), doctors who are residents, assistant medical officers (AMO) or medical officers (MO), and medical attendants (MA). Facilities refer to the zonal consultant hospital (ZCH) and the regional referral hospital (RRH), and the numbers 01-10 with the code refer to the actual FGD. All FGDs are represented by quotations.

**Table 2:** A diagrammatic interpretation of the findings

Learning features					
Educational aspects of the simulation training					
Multi-professional team training		Realism and repetition		Debriefing and reflection	
Learning outcomes					
Perceived competence			Motivational and emotional aspects		
Power of knowledge	Shared understanding and responsibility	Communication and leadership	Increased confidence (efficacy)	Reduced stress	Importance of appreciation
Confident that they save more lives					

### Learning features: Educational aspects of the simulation training

The data analysis identified three sub-categories in accordance with the first research question: Perceived effect of multi-professional team training, Learning through realism and repetition, and The importance of debriefing and reflection**.**

#### Perceived effect of multi-professional team training

Improvements in clinical PPH management were experienced after the training, and the overall emphasis within the FGDs was the importance of training in multi-professional teams during an emergency. All cadres responded positively to the composition of teams, enabling them to discuss, reflect, and value other perspectives. Being recognized as a valuable team member was a novel experience by the medical attendants, and their services were highly appreciated.

*“I am grateful on my side that those who organized the training did not discriminate against the medical attendants. Now when one shouts PPH, we also move to the site of the incident and provide our assistance.”* (MA, RRH 05).

Being trained in teams enhanced mutual understanding and allowed correction of misconceptions and recognition of the importance of everyone’s contribution. The quotation from this nurse midwife has to be read in the context of her working experiences before the training, when proper teamwork was lacking:

*“The doctors used to undermine the nurses: ‘they know nothing.’ Moreover, the nurses too felt that they were not capable of managing without a doctor. After the training, it was quite evident that nurses have a very clear role to perform, and doctors, as well as attendants. Everyone has a contribution to the team work.”* (RN/M, RRH 07).

Among medical attendants who had no direct concern for the patients before the training, the training triggered a life-saving response to emergencies:

*“The mother told me that she was bleeding. I checked her, and I found truly, it was blood. I ran at once and met a doctor who was doing rounds, I told him, ‘doctor, excuse me, please come to attend a woman who is bleeding much.’ When we came, we found really it was bleeding, and I had to shout, ‘PPH!’ That is how we rescued that mother.”* (MA, ZCH 03).

#### Learning through realism and repetition

The operator of the birthing simulator engaged all cadres of participants within the framework of their job tasks. Most of the informants described the training scenarios as realistic, with the operator of the birthing simulator performing as a real patient and mimicking the severity of PPH. In particular, the doctors from both hospitals valued the realism of the scenarios. One doctor claimed that the reality of the simulation made her think, act, and change. Another doctor described how the perception of reality made him ready to act in an emergency:

*“It was like real cases. They reached a diagnosis and called the doctor. When I assisted for the first time, I wanted to start doing something without even putting on gloves.”* (resident, ZCH 01).

Many nurse midwives discussed the importance of repetitive practice for changed behaviour. The simulation training allowed them to rehearse, repeat, and reinforce their knowledge, and thereby instantly improving their performance. Every birth is a reminder of the simulation training on how to prevent, identify, and treat PPH, and the training influenced their clinical PPH management.

#### The importance of debriefing and reflection

Through reflective learning, the informants realized that every staff member has an important role to play during emergencies, and that they could prevent maternal deaths or severe morbidity by prioritizing PPH:

*“The first training did awaken us. We did have knowledge and we were encountering emergencies, but to act quickly for an emergency and as a team, we were not that keen. But after the training, it was as if everybody understood the importance of assisting and supporting one another in saving the life during an emergency.”* (RN/M, ZCH 04).

After the training where staff got accustomed to debriefing sessions, the management of the maternity ward at the regional referral hospital prioritized the departmental morning reports to enhance reflective practice related to clinical management:

*“The staff at the labour ward does not attend the general morning report these days, but have their own morning report in the ward. They share the past days’ events…. You explain how the emergency occurred and you get contributions. We did pick this up from the training.”* (RN/M, RRH 06).

### Learning outcomes: Perceived competence

The first main category emerging from the analysis of data from the second research question on perceived competence after simulation training is illustrated by three sub-categories: The power of knowledge, Shared understanding and responsibility, and Communication and leadership.

#### The power of knowledge

Some of the informants said that their PPH management skills were inadequate. Therefore, they appreciated how the training elaborated the ABCs of the PPH protocol, being confident to undertake treatment of any PPH patient and be able to save lives.

*“Nothing brings more confidence in work than knowledge capacity building. Knowledge is power. When you know what to do, why panic?”* (RN/M, RRH 06).

Having acquired additional knowledge on PPH management and developed a shared understanding of proper PPH management within the team, a nurse midwife expressed how she perceived a demanding clinical situation:

*“The training eliminated the feeling of tiredness and stress because I am working with understanding.”* (RN/M, RRH 08).

#### Shared understanding and responsibility

All informants described the new experience of teamwork with its implications on clinical work as a major change. The doctors, especially, described the development of a shared mental model with a common understanding that enabled them to work efficiently and harmoniously within a team. Previously when a doctor came to attend to an emergency, the nurse midwife felt relieved and free to leave the doctor alone with the patient in order to attend other duties. Many of the doctors described how the burden of struggling alone with an emergency like PPH was relieved after the training:

*“We do work together. The burden which was carried by a doctor or a nurse alone has been relieved and, ultimately, our goal of saving the life of a patient is achieved.”* (AMO/MO, RRH 10).

A spirit of teamwork with shared understanding developed within the simulation training and was perceived as transferable to clinical work:

*“We are working here on the simulation and then next time we are the same team attending a patient. I can say it really increased the spirit of teamwork.”* (resident, ZCH 01).

The responsibilities of the nurse midwives in the labour ward include the allocation of patient rooms, such as admission section, labour room and operating theatre. Previously, an emergency occurring in the room next door would not require attention from others outside the room. However, after the training, the emergency was given due importance, and helping colleagues became an obligation. The difference between emergency and non-emergency was distinct:

*“Previously, we just said, ‘isn’t it only PPH?’ and we were slow. However, right now you don’t have to wait for your colleague. You jump one leg front one leg behind to get at the site.”* (RN/M, ZCH 03).

During simulation training, the attention on PPH and the need for the assistance of the medical attendants changed the way they perceived their job tasks and related responsibilities:

*“Meanwhile I continued with my business. If I was mopping, I continued to do so. However, after that training, I now know that even I am concerned, I have to serve and help the patient.”* (MA, ZCH 02).

The strength of teamwork, shared responsibility, and the relief of working together are clearly stated in the following quotation from a doctor in the regional referral hospital:

*“In the past, upon being called for PPH, you started to worry. ‘How am I going to do this?’ You were left all alone to take care of the problem. These days when a nurse calls you, you all work together, and even others are coming to help.”* (AMO/MO, RRH 10).

#### Communication and leadership

Before the simulation training, calling for help was already a part of the national and local PPH protocol. Although practiced, there was no proper response to the call for help because teamwork had not been emphasized. After the training, leadership during emergencies was established and many informants expressed how shouting for help mobilized equal attention from the team during an emergency, regardless of who was calling:

*“Now we make a loud PPH shout and everyone perceives that it is an alert sign, a life is in danger. Nowadays it sends a clear message that assistance is needed.”* (RN/M, RRH 08).

The informants realized the importance of every team member’s contribution and that a PPH emergency requires teamwork. A nurse midwife experienced how the new perception of responsibility enabled good leadership during a simulated emergency:

*“After the training, the nurse knows what to do. Now she is the pillar. She takes the position of leader.”* (RN/M, ZCH 04).

Informants at both hospitals discussed the benefits of teamwork with clear communication and leadership and the enhanced cooperation within the scope of each cadre. One of the doctors stated that the training had given them very clear guidelines. Many of the informants mentioned that the training made them aware of the importance of informing the mother and her relatives about her condition during an emergency. Before the training, they never allowed the relatives of patients to be present during emergencies and they did not inform them of the circumstances. After the training, the informants experienced that information prepared the relatives for whatever outcome, which was perceived as a relief among the informants.

### Learning outcomes: Motivational and emotional aspects

This third main category emerging from the analysis of data from the second research question is illustrated by three sub-categories: Increased confidence, Reduction in stress level, and The importance of appreciation. All informants expressed feelings of enhanced confidence and reduced stress.

#### Increased confidence

Managing PPH remained a clinical challenge after the training, but simulation training was reported to enhance the intrinsic motivation, courage, and confidence to cope with the challenges. The informants experienced their level of self-efficacy, understood as confidence in Swahili, as being closely connected to a perception of increased competence.

During the FGDs, the informants discussed how they perceived the training, including emphasis on hands-on-training on uterine massage and bimanual compression of the uterus, identification of the source of bleeding, and a focus on non-technical skills, such as communication, cooperation, and coordination. The nurse midwives expressed that they had gained competence and confidence in how to perform the life-saving procedure of bimanual compression:

*“After that training, I am courageous and confident. I can check inside up to the cervix and I am able to do bimanual compression.”* (RN/M, ZCH 02).

In the demanding situation described by a senior nurse midwife, the extraordinary was required of the entire team. A decision on whether to perform a hysterectomy (i.e., life-saving removal of the uterus) on a mother who had just given birth to her first child could only be made by the specialist. Being confident about her own judgment after the training, she decided to fetch the consultant herself by interrupting a high level meeting to get his attention. Her courage and confidence enabled a complete team to form, saving a mother’s life that day.

Previous experiences were not forgotten. Nevertheless, enhanced competence and confidence after the training made this nurse midwife certain to manage PPH, being sure that colleagues who were called upon would come to her assistance:

*“I once remember having PPH and I called for help. The person whom I called responded that if I failed to arrest it, then I could ask her. ... Training has empowered us in such way that when one shouts PPH, you will not find even a single person who is not rushing to the incident.”* (RN/M, RRH 05).

#### Reduction in stress level

Most of the informants at both hospitals shared experiences with high stress levels and low levels of confidence related to PPH management before the training. This sub-category was based on discussions of learning outcomes and how perceived stress was reduced after experiencing the value of enhanced teamwork. At the regional referral hospital, the reduced stress level in particular was emphasized, underlining the negative emotions they felt before the training. The informants described numerous demanding emergencies resulting in maternal morbidity and mortality. After being trained, their new competence enhanced motivation and reduced stress, enabling them to manage PPH timely and properly:

*“I hardly knew where to start when I encountered PPH. I always ended up in confusion. Now I know what to do, I know in more detail the causes of PPH, and most importantly I know how to manage it. Now, if I hear PPH anywhere, I don’t have that stress anymore.”* (RN/M, RRH 09).

A medical attendant expressed some of the same changes as described in the quotation above:

*“To be frank, I was very much afraid, and a diagnosis of PPH always made me shiver. But now I am strong, and when PPH is diagnosed I cooperate with others to attend and manage the patient very well.”* (MA, RRH 09).

A resident doctor used to proper teamwork after the training, missed the good teamwork while encountering an emergency during a field trip to a rural clinic. He managed to use his new teamwork skills to lower the stress experienced by the staff at the rural clinic, and to manage the emergency as a team:

*“It has decreased the stress, because when they call you, you are sure you will not be alone to manage the patient.”* (resident, ZCH 01).

#### The importance of appreciation

The informants discussed their level of motivation as an important and positive consequence of the training. They perceived that teamwork made them work as family to the patient, and everyone in maternity, the patients’ relatives, and the community should know that they worked as a team. Some informants expressed that mothers entrusting their lives to the care of the maternity staff felt relieved, knowing in their hearts that the team would do their best to manage the situation.

Nurse midwives and doctors talked about the relief of saving lives as well as the distress of losing a mother’s life. The informants knew the difficult lives of orphans, and this knowledge served as extra motivation to do everything possible to save the mother’s life. The informants shared their feelings of love among the staff and between the staff and the patients, getting on friendly terms and improving the nurse-to-patient relationship after the training. The recognition and appreciation by the families were important motivational factors:

*“When you know the steps you will take and the patient recovers from PPH and says, ‘thank you very much, sister, for what you have done!’ What else do you want?”* (RN/M, RRH 07).

Most of the informants recommended continued training within their own facilities and in all Tanzanian hospitals and health facilities in urban and rural areas to improve maternal outcomes.

## Discussion

This is one of the first qualitative studies exploring the experiences from simulation training on PPH. The purpose of the study was two-fold: to explore the informants’ experiences with learning features, and their perceived learning outcomes after multi-professional simulation training on PPH. The findings will be discussed in light of theory and previous research concerning mechanisms for learning and learning outcomes from simulation training.

Three central educational aspects of simulation training were identified: multi-professional team training, learning through realism and repetition, and debriefing and reflection. The findings indicated that team training was the overall most valuable learning feature. For the first time in these two hospitals, all maternity staff who worked together, regardless of profession, trained together. Most employees already possessed knowledge and skills before the training, and they had substantial experience. However, results suggest that the teamwork approach of the training enabled them to use their competency collectively and improve response time in demanding situations. The possibility to learn from each other’s frames of understanding and mental models and to discuss their roles and perceived responsibility in a safe environment enabled the trainees to change their own practices and how they cooperated in PPH situations. The emphasis on the importance of team training is supported by previous research. Health care providers, though well trained and medically competent, have traditionally not been trained to work in teams, where effective communication establishes and maintains a shared mental model for emergency management [43]. Findings indicate that communication with patients and their relatives was enhanced by the training and perceived as an important improvement. Teamwork comes down to timely communication, flexible coordination, and unified cooperation, and others’ lives depend on teamwork reaching a quality beyond health services provided by individuals [44].

Another finding regarding learning features accentuated by the informants as important for improvements in PPH related competence was the combination of repetitive skills training during realistic scenarios and reflective learning. Severe PPH as a life-threatening complication requires timely and evidence-based management, proper indications for interventions such as bimanual uterine compression, the knowledge of how to perform such interventions, and the confidence to actually carry out the procedure in a timely manner. Learning features, such as repetition of technical procedures with subsequent debriefing, were described to build competence and confidence. Other studies have reported the same findings. Repetitive, deliberate practice is regarded as one of the most important factors for enhanced self-efficacy [45]. Debriefing sessions for reflective learning seems to have enhanced change of mind set, enabling the informants to transfer learning to clinical practice. Many training programs have emphasized knowledge acquisition and skills training. However, our findings indicate that reflective learning can be valuable for improved practice.

Our findings indicate that the simulation training enhanced perceptions and experiences of competency level, particularly concerning the ability to manage PPH through teamwork, improved response time and team efficiency. The training was perceived to enhance a mutual understanding of roles and responsibilities, a realization that everyone has an important part to play. Many informants also expressed that participating in the simulation training had contributed to their individual competence. Enhanced knowledge of the protocol and the steps to be taken, how to perform technical procedures, and how to shout for help and communicate efficiently, made the informants recognize PPH as an important condition that could be managed successfully. The possibility of sharing information about the mother’s condition with the patients and their relatives, regardless of the severity of the emergency, stimulated by the simulation context, was perceived to relieve stress. After the training, nurse midwives and medical attendants felt empowered and motivated to take responsibility in a PPH related emergency, where they had previously avoided participation. These findings are in line with previous studies. The most valued factors in team training among peri-operative staff have been reported to be communication, cooperation, and coordination, together with situational awareness and leadership [46]. Another study demonstrated that, if different health cadres train separately, it can be difficult to integrate their competencies into a team [22]. Skills are important, but teamwork skills are crucial, knowing that failures in communication and teamwork are major causes of adverse obstetric events [43, 47].

A motivational and emotional aspect was another category reflecting training outcomes. The informants experienced a significant influence of the training on the motivational and emotional aspects of handling PPH*.* Many of the informants expressed a higher level of self-efficacy after the training because they felt more knowledgeable and confident about PPH management. Improvements in perceived PPH related self-efficacy are likely to be important for the ability to perform necessary skill in PPH emergencies. Even health care providers who have the necessary knowledge and skills to manage a certain emergency, can fail unless they have adequately strong self-efficacy [48]. Perceived self-efficacy has been found to increase significantly with high-fidelity simulation training in obstetric emergencies [49], and increased self-efficacy has also been found to be associated with improved performance [50].

Our findings also suggest that the focus on working with understanding and confidence in team support reduces the feeling of stress. The mastery experiences during scenarios, and later in clinical practice, were described as contributing to a significant reduction in stress levels and increased confidence. The informants’ previous negative experiences were shared frankly and discussed during the FGDs. Some of these experiences were also shared at the very beginning of the simulation training, and paved the way for frank communication about negative as well as positive experiences. The contrast between previous traumatic experiences and the strengthened confidence level after training, was a positive outcome of the study.

Positive changes in PPH management were also recognized by the mothers giving birth, their relatives, the community, and the regional health authorities. Community appreciation was perceived as very important and a strong motivational factor for the provision of health care, as well as recognition by the health authorities. Community appreciation is one of the key sources of encouragement among Tanzanian health care providers [51] and an important indicator of the quality of provided services. The staff at the regional referral hospital experienced to be given a highly appreciated award for proper PPH management after an inspection visit from the Ministry of Health half a year after the first training.

The findings related to the importance of compassion cultured through team training, both among the staff and between the staff and patients, is supported by other research [52], demonstrating that compassion among co-workers is associated with work satisfaction and teamwork, whereas negative relationships cause emotional exhaustion.

Team composition reflecting authentic sociological fidelity was perceived as an important learning feature [53]. The challenge of hierarchy and power relationships was addressed during the debriefings and reflected in the discussions in statements about the importance of teamwork, regardless of position and years of experience.

### Strengths and limitations of the study

Some strengths and limitations of the study should be noted. The FGDs consisted of groups of nurse midwives and medical attendants together, and groups of doctors only. This was done for convenience but did not reflect the multi-professional teams that trained together.

However, this balanced focus group schedule may have helped everyone to have a voice.

Another limitation was the lack of an observer’s participation in the FGDs. An observer could have sensed the atmosphere during the discussions and taken notes, enabling latent content analysis.

The informants did not share negative perceptions related to the simulation training during the FGDs, and it is timely to ask whether the threshold for expressing criticism was generally high in this setting. The fact that they knew the interviewer as a part of the research team during simulation training might have prevented them from expressing negative perceptions. The straight forward way of sharing information about previous negative experiences indicated that the informants felt free to express positive and negative experiences and feelings. Through reflective practice and honest feedback, they experienced change of mind set and a shared understanding of PPH management. The frank communication throughout the debriefing sessions was considered a strength of the study. The combination of having a burdensome working situation and participation in simulation training perceived as highly relevant, may explain the overall positive experiences from the training. A closing question asking for any additional comments from the informants did not reveal any negative perceptions, only demands for more training.

## Conclusions

Multi-professional simulation training on PPH in two Tanzanian hospitals was experienced as highly relevant. The findings from this qualitative study suggest that training in teams, skills training and realistic scenarios, repetition of scenarios with subsequent debriefing for reflective learning, were crucial for enhanced teamwork. Informants perceived improved relationships through improved communication and situational awareness. An increased acquisition of competence throughout the training, could be implemented the next day on duty.

The perceived results of the training enhanced confidence and were most likely influenced by the systems approach to human error. Acknowledging that human nature is fallible and human error is primarily a consequence of working conditions and not someone’s carelessness or negligence, most likely reduced the individual pressure and risk of personal blame. The informants shared numerous negative experiences with severe PPH as being a killer before the training, with memories of stress, panic, and guilt. However, communication with patients and their relatives throughout the emergencies reduced the feelings of guilt and stress. The acknowledgement and appreciation of improvements in PPH management from mothers, their relatives, the community, and the health authorities, together with compassion for colleagues through teamwork, were highly motivating factors.

### Implications of findings for practice and future research

There are few qualitative follow-up studies after training programs in obstetrics. Allocating staff for training is known to be resource demanding, and qualitative research is needed in low- and high-resource settings to ensure that educational interventions are relevant, effective and sustainable, resulting in improved patient safety. Many training programs emphasize hands-on training on technical skills without allocating time for reflective learning in teams. Based on our findings, team training should include debriefing sessions run by qualified facilitators to stimulate reflective learning. Further research is needed to explore the optimal educational design of simulation training on obstetric emergencies like PPH.

## Appendix 1

**Table 3:** Interview guide used for focus group discussions at both hospitals

1.	Can you elaborate how the simulation training contributed to change in confidence level regarding PPH-management?
2.	Can you elaborate how the simulation training influenced how you consider the importance of managing PPH?
3.	Can you explain how the simulation training influenced your way of working as a team?
4.	Please share experiences of events where you used knowledge and skills learned from the training.
5.	Can you describe any change in how you perceive being responsible for a mother developing PPH?
6.	In which way has the training affected your perception of calling for help?
7.	In which way might the training have benefited the patients?

## Abbreviations

AMO: Assistant medical officer
FGD: Focus group discussion
MA: Medical attendant
MO: Medical officer
PPH: Postpartum hemorrhage
RN/M: Registered nurse/midwife
RRH: Regional referral hospital
ZCH: Zonal consultant hospital
